# What is traditional acupuncture - exploring goals and processes of treatment in the context of women with early breast cancer

**DOI:** 10.1186/1472-6882-14-201

**Published:** 2014-06-25

**Authors:** Sarah Price, Andrew F Long, Mary Godfrey

**Affiliations:** 1 The Complementary and Integrated Medicine Research Unit, Faculty of Medicine, University of Southampton, Southampton SO16 5ST, UK; 2 School of Healthcare, University of Leeds, Baines Wing, Leeds LS2 9UT, UK; 3 Leeds Institute of Health Sciences, University of Leeds, 101 Clarendon Road, Leeds LS2 9LJ, UK

**Keywords:** Acupuncture, Qualitative, Breast cancer, Complex intervention, Model validity, Practitioner practice, Process-outcomes

## Abstract

**Background:**

Despite the increasing popularity of acupuncture, there remains uncertainty as to its effectiveness and how it brings about change. Particular questions are posed over whether acupuncture research has sufficient model validity and reflects acupuncture as practised. Exploring traditional acupuncture (TA) in practice should help to expose processes essential to the theory of TA. The aim of this study was to examine what TA practitioners aim to achieve, their rationale and how they follow this through in their practice.

**Methods:**

A longitudinal study of TA for women with early breast cancer (EBC) was performed. Study participants comprised 14 women with EBC and two experienced TA practitioners, all taking part in in-depth interviews, conducted before and after receipt of up to 10 treatment sessions, and analysed using grounded theory methods. Additional data came from practitioner treatment logs and diaries.

**Results:**

Practitioners sought long-term goals of increasing strength and enabling coping as well as immediate relief of symptoms. They achieved this through a continuous process of treatment, following through the recursive and individualized nature of TA and adjusted, via differential diagnosis, to the rapidly fluctuating circumstances of individual women. Establishing trust and good rapport with the women aided disclosure which was seen as essential in order to clarify goals during chemotherapy. This process was carefully managed by the practitioners and the resultant therapeutic relationship was highly valued by the women.

**Conclusion:**

This study provided insight into the interdependent components of TA helping to demonstrate the multiple causal pathways to change through the continuous process of new information, insights and treatment changes. A good therapeutic relationship was not simply something valued by patients but explicitly used by practitioners to aid disclosure which in turn affected details of the treatment. The therapeutic relationship was therefore a vital and integral part of the treatment process.

## Background

Acupuncture is a diverse and global practice and an increasingly popular complementary therapy. However, there remains uncertainty as to its effectiveness as well as how it brings about change. In particular, questions have been posed as to whether acupuncture research has sufficient model validity and reflects acupuncture as practised [1-3]. If there is insufficient model validity, the effects found within research may not be reproduced when acupuncture is delivered in practice.

In order to understand further the theory of how acupuncture effects change in research and in acupuncturists’ practice, insight is needed into what practitioners aim to achieve, why and how they follow this through in their practice. The picture is complicated by the use of different theoretically-driven approaches to acupuncture and acupuncture practice, in particular, medical acupuncture (MA) or traditional acupuncture (TA) [4]. Alongside, there is increasing evidence that these approaches are experienced differently by participants and perceived to achieve different outcomes [5]. To explore these issues further, this paper presents findings from a longitudinal, qualitative study of the receipt of TA for women undergoing chemotherapy as part of their treatment for early breast cancer (EBC). The paper aims to provide deeper understanding of practitioners’ goals and how these are addressed through their practice delivery of TA.

### Background: practitioner and patient perspectives

Underlying TA theory points, in particular, to a focus on the holistic whole-person in their life world, individualized diagnosis and treatment [2]. There is however only a small corpus of qualitative research that examines how TA practitioners follow this through in their practice and in their overall treatment goals [6-8]. Each study involved semi-structured interviews with practitioners, all practising TA and members of the UK professional accreditation body, British Acupuncture Council (BAcC) whose members predominantly practise TA. Hughes et al. [7] also interviewed practitioners of ‘*western acupuncture’* in order to explore contrasting approaches to practice. MacPherson et al. [6] employed a nested qualitative study within a large RCT for chronic low back pain. In contrast, Jackson and Scambler [8] examined practitioners’ attitudes to scientific evidence and how this influenced their practice.

While practitioners’ goals varied in relation to the client group, common elements were visible: symptom reduction; and enablement of broader health, well-being and lifestyle effects. For example, in their study of patients with chronic back pain, MacPherson et al. [6] highlighted an overall goal of long-term improvement in the symptoms patients experienced, coupled with a goal of enhancing patients’ understanding and attitudes, to effect change in self-care. Both of the other studies [7,8] pointed to improvement in symptoms, along with improved general physical and mental health and increased emotional well-being.

These goals were achieved in two main ways: the process of individualised diagnosis and pursuit of a holistic approach; and, development of a strong therapeutic relationship, thus engaging the patient with treatment, supporting her/him to gain insight into the link between their illness and broader life experiences and enabling ‘*increased agency in self-*care’ [9]. Such co-working would likely also lead to changes in treatment goals [7]. All three studies emphasised the holistic nature and individual focus within treatment, with MacPherson et al. [6] suggesting the possibility of involving multiple causal pathways. Changing behaviour was seen as the lynchpin in obtaining long-term goals of improvement in the various parameters of chronic illness experience.

Practitioners used the explanatory model of traditional Chinese medicine (TCM) to link the patient’s illness experience to other broader life circumstances, both in terms of understanding illness onset and how it might contribute to improvement or deterioration [6] as treatment progressed. They were thus *‘helping patients make sense of their condition’* within a Chinese medicine explanatory model. The practitioners also talked about the different ways that TA worked through assessing the strength of the individual’s *Qi,* regulating *Qi* and addressing energetic imbalances*.* It is worth noting that a fundamental concept in TCM or TA is *Qi;* there is no equivalent concept in western culture, but it is often translated as ‘energy’. The literature suggests that TA ‘works’ by effecting change in the balance of Qi, Yin and Yang and other imbalances as defined by the differential diagnosis [4,6-8].

Further insight is provided from qualitative studies of patient experiences of TA. Cassidy [10], in a large US survey exploring what users of TA valued about their treatment, pointed to two levels at which change occurs: those related to symptoms; and those pertaining to the whole person, including new holistic understandings. Similar findings arose in Gould and MacPherson’s UK study [11], with participants also expressing appreciation of an *‘educative’* style of treatment. This was echoed in a phenomenological oriented study involving 12 clients in an Australian setting [12]. Here, participants expressed their sense of being cared for because of the wide ranging issues considered in the differential diagnosis which conveyed a sense of real interest in them as an individual.

Other qualitative studies [9,13-15] provided insight into patient perspectives over how the treatment might work. In Paterson and Britten’s UK study [13], participants discussed their treatment not just in terms of needling skills but also in relation to the therapeutic relationship and their developing ‘*new understanding of the body and self as a whole being’*. Hughes et al. [14] found patients who were treated by a TA practitioner were more likely to describe the treatment as encompassing more than needles and to effect whole person changes that enabled them to regain their lives. Again, in Rugg et al’s sample of 20 patients [15] nested in a RCT of treatment for medically unexplained symptoms, participants pointed to their being actively engaged in the treatment process, the practitioner listening and responding to them. They felt involved because the practitioners asked them many questions, which led to their negotiating or considering making changes to their diet, relaxation, exercise and other social activities, and thus increased agency in self-care. Evans et al. [9], in a study focusing on self-care talk, involving audio-recordings of 21 consultations and subsequent telephone interviews with 18 clients, showed how self-care talk was individualized by embedding it within the differential diagnostic process. Moreover, the therapeutic relationship and communication facilitated this increased agency in self-care.

In summary, current literature, from both a practitioner and patient perspective, draws attention to the whole person approach, the differential diagnosis and the therapeutic relationship. One of the core long-term practitioner goals was to bring about change in behaviour using the explanatory model of TA to address imbalances in the *Qi* via the diagnostic and treatment processes.

### Early breast cancer

Existing qualitative literature on TA is primarily concerned with long-term conditions. Studying the approach of TA practitioners, and the experiences of their clients in an acute situation might add further insight into the goals sought and how these are achieved. Such a case is provided by a recent diagnosis of early breast cancer (EBC). Breast cancer is the most common cancer in women, accounting for nearly one third of all new cancers for women. In the UK in 2010, there were 11,556 deaths from breast cancer and a woman has a one in eight chance of a breast cancer diagnosis in her lifetime [16]. As breast cancer treatments have extended over months and in some cases years, it may be experienced at different points as chronic interspersed with acute episodes, requiring multiple, intensive interventions [17]. Women diagnosed with breast cancer are likely to very quickly undergo surgery, then chemotherapy followed possibly by other adjuvant treatments such as Herceptin, radiotherapy or Tamoxifen. The speed of the diagnostic and initial treatment phases in the breast cancer trajectory reflects evidence on the significance of early diagnosis and treatment on increasing survival.

### Aim

The aim was to further explore what TA practitioners seek to achieve and how they follow this through in their practice with women with EBC who are going through their bio-medical treatment. A variety of qualitative data collection methods (interviews, diaries and treatment logs) were used to enable detailed insight into change over time.

## Methods

A longitudinal study was developed and conducted within a real world setting [18], offering women who had recently been diagnosed with EBC the opportunity to access, via their oncologist, up to ten sessions of free TA while they underwent chemotherapy. Fourteen women with EBC were recruited from two NHS hospital trusts in the UK; sample size was restricted due to funding constraints and the need for intensive in-depth interviews undertaken over time. Two experienced acupuncturists provided the TA sessions, at one of four centres, including one private practice, and were asked to approach treatment as they normally would, thus replicating real world practice. The acupuncturists were members of the British Acupuncture Council, the accrediting body for TA in the UK. The acupuncturists were invited to the study because of their experience in practice; both had been clinical supervisors at an accredited college and they were also willing to participate.

In-depth interviews, lasting between 50 and 120 minutes, were conducted by SP, recorded and fully transcribed. All participants were given a pseudonym. The women were interviewed before the start of chemotherapy, during chemotherapy (at around the 5^th^ chemotherapy infusion) and, for half of the study group, approximately six months after chemotherapy had finished (although three of these were yet to embark on a further 12-months of three-weekly hospital based Herceptin infusions). Theoretical sampling was used for the third interviews, to explore whether the meaning and significance of the treatment journey varied with life stage (transitioned to retirement, unable to return to work or resumed previous work) and to capture change over time. All the interviews took place at two of the complementary therapy centres, with one exception, which took place at hospital. Interviews started with an open question (for instance, in the first interview: “please tell me how you discovered you had breast cancer?”) and continued in a conversational form, led largely by the participants’ responses. An interview guide was used to ensure that all areas were covered by the end of the interview.

The practitioners were interviewed (by SP) before and after completion of all TA treatment with participants, and they kept treatment logs and diaries. Treatment logs provided in-depth understanding of their reflections on what they did and why, a contemporaneous picture of their actual practice with individual women and any new problems experienced by their patients which resulted in altered treatment priorities. Their diaries offered reflections on treatment related issues.

Data were analysed using a grounded theory approach (led by SP). This included simultaneous data collection and analysis, open coding, and memo-writing, leading onto focussed coding and the development of key categories [19] using the method of constant comparison [20]. Field notes were made immediately after each interview including methodological, observational and reflexive notes. As SP is an acupuncture practitioner, it was important that a reflexive stance was maintained throughout the study. The diaries and treatment logs were included in this process. The on-going analysis was checked for inclusiveness and consistency with co-authors, and the data searched for disconfirming cases. As befits a grounded theory approach [21] focus lay on process and change. For example, attention was directed at understanding how practitioners assessed and responded to women’s articulation of symptoms and experience of illness as these changed over time and in relation to the treatments, and how women in turn experienced acupuncture in the context of the meaning and experience of breast cancer at this stage in the illness trajectory.

Written informed consent was obtained from all participants who were told they could withdraw from the study at any time. All data has been anonymised. Ethical approval was obtained from Leeds (East) Research Ethics Committee (REC approval number reference: 07/H1306/79). The findings relating to how TA is practised and how treatment processes related to goals are reported here whereas the findings relating to the perceived benefit of TA have been published elsewhere [22].

## Results

The mean age of the women was 54 (range of 41–76). Six had dependents, either elderly parents or children under the age of 16 living at home. All but one of the participants had a household income of less than £30,000 and two had a degree or equivalent educational status. All of them had surgery (two had a mastectomy) and all received six cycles of EPI-CMF chemotherapy, begun just before their receipt of TA. Thus, at the onset of the study and their first meeting with the TA practitioners, the women were both struggling with coming to terms with the diagnosis of EBC and experiencing symptoms resulting from their medical treatment that were variable, changing from day to day and over time. Moreover, the women were also at a point in the illness trajectory when they still hoped to resume a ‘normal’ life while simultaneously fearful of the longer-term impact of EBC and the threat it posed for a shared future life with those close to them. These were thus all issues that the practitioner needed to understand and recognise in taking forward TA with the women.

### Practitioners’ intended outcomes

Uncovering what the practitioners were trying to achieve is an important step in backwards mapping the processes that facilitate perceived or desired change. Intended outcomes related to two categories of change: immediate relief of symptoms which were changing from day to day; and, for the longer term, enabling coping. Enabling coping was believed to be brought about partly by relief of symptoms and partly by increasing *strength* in the face of suffering.

Diane (practitioner 1) talked about dealing with the issues that were important to the person and to get the individual to a point where they were strong enough to cope. Helen (practitioner 2) indicated that by supporting the person through easing of their main symptoms or concerns they were more able to cope. Both practitioners used the language of TCM in discussing what they were trying to achieve. In this study, enabling coping was both a result of strengthening and fortifying the person’s *Qi*, but also through alleviating other symptoms, for instance, pain, insomnia or anxiety.

For EBC sufferers where fatigue and tiredness were daily experiences, increasing vitality was of paramount importance to the practitioners. ‘*Strength*’ was about the whole person, and not just physical strength, including, for example, emotional strength to deal with difficulties. Addressing the fear and anxiety from the life threat were seen as the target for strengthening the woman emotionally.

*‘You lose your ability to cope if you’re emotionally distressed. So to me to help her feel stronger would help her to deal with the chemotherapy and whatever life is going to throw at her with the treatment*’. Diane

Enabling coping and wellbeing were important desired outcomes. The practitioners considered how they were to achieve this in providing treatment, seeing it as achievable mainly through the acupuncture needling and partly through talking. Helen explained that coping was linked with tiredness and that the breast cancer sufferer’s ‘*energy*’ was of principal importance.

*‘You know whatever else is going on in her life I think that would be one of my main worries really (energy); you know can I get to grips with the tiredness, because it will be a principal issue because she can’t cope if she is tired and not sleeping*’. Helen

The practitioners used the language of ‘*fortifying’, ‘increasing the vitality’* and *‘tonifying* the *Qi’* of the person; this was a key part of their aim of strengthening and balancing the whole person. By strengthening and fortifying the *Qi,* the person is more able to withstand emotional distress and also manage physical discomfort.

The treatment logs provided further insight. These showed that practitioners changed their treatment plan regularly depending on what new and pressing symptom was presenting, while at the same time continuing to address more long standing issues such as fatigue or night-sweats. For example, according to Diane’s log, one woman, Jane, experienced varying symptoms during her ten sessions of acupuncture, with fatigue always being in the background, but at times suffering mental distress and depression over the cancer returning, and a multitude of aggravating daily symptoms (Table 1). From the diaries, the practitioners expressed treatment dilemmas in terms of whether focus should lie on immediate relief of symptoms or longer term goals and the balance in between. These data provide evidence of a reflexive approach to treatment, constantly weighing up and preparing to change direction depending on what new information was available.

**Table 1:** Extracts from treatment log (for Jane) by Diane

**Treatment number**	**Main concern**	**Second concern**	**Comments by practitioner**
1	Stress	Worry about immune system and fear of recurring infection	Was hospitalised (weeks) after breast op with infection
2	Fatigue	Poor sleep, reflux and headache	More relaxed after treatment 1
3	Sore mouth no taste	Fatigue	No headaches, sleep and reflux improved

### Processes related to achieving goals

In order to achieve the immediate and long-term goals, further insight comes from looking at the process of practitioners’ treatment, that is, the way they facilitated change. Two core themes emerged from the data, the continuous process of treatment and the dimensions of the processes of treatment that this embraced.

### Continuous process of treatment

**Figure 1:**
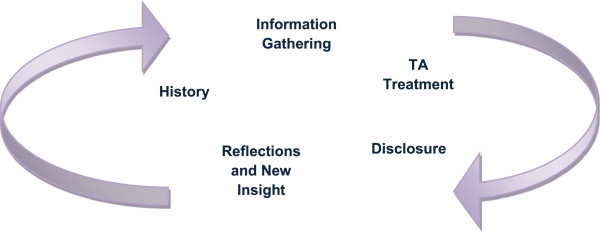
The continuous interlinking processes of TA delivery.

Different dimensions of the TA treatment were encompassed by this continuous process. The practitioners spent time explaining how *gathering information* was crucial to the differential diagnosis (Bian Zheng), a core part of TCM practice. The practitioners both explained that developing a good therapeutic relationship was critical in order to get as much information as possible, to see the whole person in the context of their lives, and to understand their needs and concerns. The acupuncturists relied on *disclosure* undertaken via developing this relationship, enabling a *continuous process* of new information, new insight and understanding and changed *treatment* (see Figure 1). TA ‘worked’ through the process of diagnosis and on-going disclosure (both backward and forward looking) facilitated by the therapeutic relationship, leading onto new practitioner insights assisting them to reform/modify their diagnosis and change the treatment.

Information for the practitioners was not always wholly reliant on the relationship as they also discussed *‘silent information’*, and this fed into their analysis of the whole situation. Silent information was what they observed of the person in terms of their appearance and presence. For instance, in TCM theory, the strength of a person’s voice or handshake provides some information about their *Qi.* Other diagnostic information gathering, such as pulse-taking, involved touch but not necessarily talking.

The *history* of the person, not just the aetiology of their symptoms, was also of interest, as practitioners looked at these women in the context of their past as well as how they expressed their desires for the future. An example of this is a reflective comment in Helen’s diary, where she discusses Lena, and her history of working nights full time in what by anyone’s perspective would be a stressful job, along with bringing up her 3 children and caring for her father. *‘Of particular note was the very pressured life she had prior to diagnosis’.* Helen reflects on how Lena presents according to her *differential diagnosis* at the first treatment (and prior to chemotherapy starting) and ponders on what impact the chemotherapy might have on her:

“What am I trying to achieve? To strongly nourish her basic deficiencies of Kid Yang and Yin in order to minimise effects of chemo. Vigilance on 1) already v. yin xu – chemo may burn yin easily – current hot flushes could worsen/ stomach may become yin xu.…”

This TCM diagnosis of ‘yin xu’ can be translated in lay terms as meaning that the individual presents with a substantial imbalance and deficit in their system. In this case this is shown through the signs and symptoms of hot flushes and digestive problems. Helen is expressing concern over the impact chemotherapy will have on this already presenting deficit. The diary entry continues with the conflicting needs, in terms of how to approach the TA treatment.

Interviews with Lena attest to this problem. In her second interview during her treatment with chemotherapy, Lena highlighted that hot flushes and night sweats were a major problem; at one stage she described her head as *‘steaming’*. In her third interview, three months after the end of her chemotherapy treatment, Lena describes really having missed the TA as her health, especially her digestion, had severely deteriorated during the subsequent months of chemotherapy without TA.

When asked what has to change to alter their treatment, Helen commented:

*‘I suppose it is on different levels all the time. Part of it is just hopefully a progression you know, you’ve made a decent diagnosis, you’ve done the decent treatment, and the person is progressing. So the treatment has to follow the patient’s progression and will change as the person’s symptoms change*’. Helen

Over the course of the treatments and changing symptom experiences, the women’s priorities altered or they revealed information that modified how the practitioner made the diagnosis or the practitioner had new insight, so treatment was changed. The following extract from Diane’s diary illustrates this:

“Two new ladies today. Lynne was very sensible and sorted in her head, so she thought, and said she wasn’t in denial. However, once the needles were in she cried and was embarrassed by this. We talked quite a lot about what had happened. Hopefully she sees it as a positive sign that everything is releasing”.

Both practitioners talked of a continuous treatment process. It was an important element in the drive to quickly establish good rapport and trust so that they could find out early on what the real issues were; aiding *disclosure* (a dimension of this process) was a priority which was dependent on the developing therapeutic relationship. Figure 1 above displays the different dimensions of the processes of TA delivery encompassed by this continuous process. Information was gathered slowly over time, as different strands of this continuous process were woven together.

Change was anticipated by the practitioners from the acupuncture treatment, from talking, and from patients’ reflection over time. Building rapport and trust and having a framework for enquiry was viewed as giving rise to more in-depth disclosure; the acupuncture treatment itself facilitated relaxation and trust, reported by both the practitioners and the women. As women experienced immediate benefit from the TA, their trust in the process increased. Over time, and with each treatment, more in-depth knowledge of the individual was discovered by the practitioner. This did not preclude new insights coming from the women participants also. Disclosure and reflections with new insight were process-outcomes for the practitioners and fed into new information and new treatment, as the following extract from Diane’s second interview, conducted once all the TA sessions with the women were completed.

*“Well, when they come in and they are talking twenty to the dozen, and they can’t get hold of a thread to have a conversation through so we can go from a to b to c.... They are all over the place…. very agitated, physically agitated, can’t sit still. And I find that after a session of acupuncture, if they are like that, and you can use some points … so they are feeling more – I can’t say better than grounded – instead of the Qi all whizzing around in their head, all head stuff, not knowing what to do with it. It gives them some breathing space, so it gets the Qi circulating, nice and calmly, grounds them so that they can actually get some space in their head, to be logical again*”. Diane

### What women valued about the process of TA care

The achieved outcomes and perceived benefits the women experienced from TA have been reported elsewhere [22]. Here it is important to focus on the perceptions of benefit related to the processes of treatment. This provides further insight and importance to the notion of a continuous process of treatment that changed over time.

While the women felt better from the acupuncture in ways that seemed difficult for them to articulate fully, they placed considerable value on aspects of the treatment process. Feeling immediate relief of symptoms seemed to increase their belief in the TA, which engendered trust in the process and consequently in the practitioner. Some of the women reported that lying on the treatment couch and relaxing led them to chat more openly with the practitioner. Only two women had previous experience of acupuncture from a physiotherapist (back pain and neck pain); and all had very little idea of what to expect or what it might do for them in respect of their EBC. Disconfirming cases related to whether acupuncture could be responsible for change, more than whether participants valued and enjoyed the process of acupuncture care.

The main property of the treatment process that the women valued was the therapeutic relationship. Key dimensions included the practitioner listening and paying heed to what was said, the rapport established and a perception of the practitioner as offering both a friendly and supportive presence and providing space to talk about oneself. Even though the conversation was often described as ‘*light and chatty*’ this relationship was highly valued, and for some, described as of equal importance to the perceived benefits from the acupuncture treatment. Characterising the therapeutic relationship is beyond the scope of the aims of this paper; what is important to note is that while the practitioners used the relationship to aid disclosure, the women valued it as an outcome in itself.

## Discussion

This longitudinal study has provided further insight and clarification of how practitioners went about achieving their goals in their practice of TA in relation to treating women while undergoing chemotherapy for EBC. Clarification of the practitioners’ goals, reported in a previous paper [22], pointed to the importance of seeking and actively pursuing immediate relief of symptoms and, for the longer term, enabling coping and increasing strength. Alongside, the women [22] experienced and valued benefits of their TA from the general (ranging from feeling more balanced to being more peaceful and energised) and the specific (symptom relief). The findings presented in this current paper point to ways in which these outcomes were achieved. Foremost, and not previously demonstrated in other studies, was the continuous process of information gathering, treatment, disclosure, new insight and reflection. Such a finding also adds weight to the notion that TA is a complex intervention with multiple causal pathways.

In comparing the findings from this study with earlier qualitative studies for TA, one unique difference is that the experiences of the women participants, all with recently diagnosed EBC and symptoms from chemotherapy, were recent and acute, not chronic, including the shock and distress of diagnosis and what breast cancer might mean in the longer term. In contrast, other qualitative studies of TA have focussed on treatment of chronic symptoms experienced within long-term conditions.

In the current EBC study, a core and critical dimension of the therapeutic relationship, and its main focus for the practitioners, was to aid disclosure to enable better treatment aligned to clear goals. This aided the practitioners in achieving their treatment goals. In common with other research [6-9,15], developing a good therapeutic relationship was critical. But in this study it was being used for a different purpose; previous TA studies pointed to it being more used to engage patients with the TCM notions of illness and thus bring about changes in behaviour and lifestyle. Both uses helped TA recipients gain new insight and understandings of their illness and enable increased agency in self-care.

In our EBC study, it was evident that TA operated in recursive ways to effect change in the shorter term. This was very important for the women; they were very concerned at the onset and during chemotherapy, with the *‘here and now’* and getting through or coping better, rather than benefits of TA over the longer term; in particular, nearly all participants expressed regular worry about their long-term future. The significance of the nature of the client-practitioner relationship and its particular nuance here may be specific to the acute phase of the illness that participants were going through, in contrast to the chronic phase which forms the focus of much existing TA literature. The emphasis and significance of the therapeutic relationship within TA may thus vary depending on the phase of a potentially life threatening condition. For these women, the experience of the acute phase was centred on the shock of diagnosis and emotional distress as well as the physical effects of treatment.

The work also demonstrates and provides insight into TA’s possible multiple causal pathways. Multiple causal pathways make the division between process and outcome ever more blurred, as suggested in other TA literature [1-7]. This has implications for research design. TA is experienced as person-centred and responsive to change by recipients and in its delivery over time. Setting up a longitudinal study, albeit with a small sample size, to observe change over time offered rich participant data providing additional insight into what acupuncture is. In particular, the ‘before and after’ practitioner interviews enabled rich data and insight over time as to how the practitioners thought about their treatment approach and how they did it, relating parts of their explanations to specific patients. The findings from these data are further strengthened by the use of triangulated methods, utilising interviews, diaries and treatment logs. Limitations of this study include the small sample size and the fact that data was gathered from two practitioners.

## Conclusion

This study provided insight into the interdependent components of TA helping to demonstrate the multiple causal pathways to change, through the continuous process of eliciting new information, formulating, implementing and reviewing treatment strategies. In so doing, it has provided insight into how TA brings about change in a set of women experiencing acute symptoms of EBC at an early phase in their illness trajectory. A good therapeutic relationship was not simply something valued by patients but was explicitly used by practitioners to aid disclosure which in turn affected details of the treatment. The therapeutic relationship was therefore a vital and integral part of the treatment process. The findings lend further weight to the conception of TA as a complex intervention.

## Abbreviations

EBC: Early breast cancer; TA: Traditional acupuncture; TCM: Traditional Chinese medicine.

## Competing interests

The authors declare that they have no competing interests.

## Authors’ contributions

SP conducted the original literature review that informed the study, conducted the interviews and drafted the original manuscript. Analysis was undertaken by all three authors. All authors read, and contributed to editing of the article and approved the final manuscript.

## Pre-publication history

The pre-publication history for this paper can be accessed here:

http://www.biomedcentral.com/1472-6882/14/201/prepub
